# Deep Learning-Based EEG Emotion Recognition: A Review

**DOI:** 10.3390/brainsci16010041

**Published:** 2025-12-28

**Authors:** Yunyang Liu, Wenbo Xue, Long Yang, Mengmeng Li

**Affiliations:** 1 School of Electrical and Information Engineering, Zhengzhou University, Zhengzhou 450001, China; liuyunyang@stu.zzu.edu.cn (Y.L.); xwbzzu@gs.zzu.edu.cn (W.X.); longyang_zzu@163.com (L.Y.); 2 Henan Key Laboratory of Brain Science and Brain-Computer Interface Technology, Zhengzhou 450001, China; 3 State-Owned Luoyang Dancheng Radio Factory, Luoyang 471000, China

**Keywords:** EEG, emotion recognition, deep learning, neural networks, attention mechanism, transfer learning

## Abstract

Affective Computing and emotion recognition hold significant importance in healthcare, identity verification, human–computer interaction, and related fields. Accurate identification of emotion is crucial for applications in medicine, education, psychology, and military domains. Electroencephalographic (EEG) signals have gained widespread application in emotion recognition due to their inherent characteristics of being non-concealable and directly reflecting brain activity. In recent years, with the establishment of open datasets and advancements in deep learning, an increasing number of researchers have integrated EEG with deep learning methods for emotion recognition studies. This review summarizes commonly used deep learning models in EEG-based emotion recognition along with their applications in this field, including the design of different network architectures, optimization strategies, and model designs based on EEG signal features. We also discuss limitations from the perspectives of commonality–individuality (C-I) and suggest improvements. The review outlines future research directions and provided a minimal C-I framework to assess models. Through this review, we aim to provide researchers in this field with a comprehensive reference and approach to balance universality and personalization to promote the development of deep learning-based EEG emotion recognition methods.

## 1. Introduction

Emotions are an important component of human psychology and behavior, and their recognition and analysis have significant theoretical and practical value in various fields such as psychology, neuroscience, human–computer interaction, and intelligent health. Emotion recognition tasks can be based on a variety of different types of signals. Emotion recognition based on facial expressions or speech, although intuitive and easy to collect, is easily disguised. Emotion recognition based on physiological signals such as electroencephalogram (EEG), electromyography (EMG), and electrodermal activity (EDA) has received increasing attention due to its difficulty in being disguised and its inclusion of more informative characteristics.

In recent years, with the rapid development of brain–computer interface technology, EEG, as a physiological signal that can directly reflect brain activity, has gradually become an important tool in emotion recognition research. EEG signals have the advantages of high temporal resolution, non-invasiveness, and real-time monitoring, which can capture subtle changes in neural activity related to emotions and provide strong support for a deeper understanding of the neural mechanisms of emotions.

However, the complexity of brain signals, individual differences, and the multidimensional and dynamic nature of emotions pose significant challenges for EEG-based emotion recognition. A key issue involves identifying commonalities and individuality in participants’ emotional EEG patterns. Commonality refers to shared patterns of specific emotional states across individuals, while individuality denotes distinct features between participants, stimuli, or datasets, which are associated with individualized experiences, personality traits, equipment usage, etc.

Traditional signal processing and machine learning approaches exhibit limitations in feature extraction, model generalization capabilities, and the accuracy of affective classification. In recent years, the rapid advancement of deep learning technology has brought new hope for addressing these challenges. Various deep learning algorithms, leveraging their powerful automatic feature extraction capabilities and ability to model complex patterns, combined with the attention mechanisms and transfer learning techniques that have gained popularity in recent years, are gradually becoming a research hotspot in the field of EEG-based emotion recognition.

Existing reviews mainly focus on the latest deep classification models and algorithms, pay less attention to the fusion modeling of individual differences and common features in emotion recognition, and lack of in-depth discussion on cross-domain generalization ability, and also lack of a model to evaluate what and why new methods might be affective, which limits the application effect of relevant methods in practical scenarios. We proposed several questions and tried to find the answers:

Q1. What is the history and current situation of deep learning methods in emotion recognition?

Q2. What strategies can be applied to improve subject-independent experiments (more similar to real-life applications with few or no previous training data)?

Q3. From what perspectives can we explain any improvements?

This article systematically explores model development and recent research advancements in the field of deep learning-based EEG emotion recognition from the perspective of commonality and individuality. First, we review the fundamental principles of EEG-based emotion recognition and the limitations of traditional methods. Subsequently, we provide a detailed introduction to deep learning models and their applications in this domain, with particular focus on how these models identify common emotional patterns in EEG signals and explore leveraging individual differences to enhance model performance. This includes discussions on different network architectures, optimization strategies, model improvements based on EEG signal characteristics, as well as comparisons of limitations and enhancements. We also outline future research directions. Through this review, we aim to provide researchers in this field with a comprehensive reference framework to facilitate further development of EEG-based deep learning emotion recognition technologies.

## 2. Literature Search and Selection Methodology

### 2.1. Search Strategy

To establish a comprehensive and transparent foundation for this narrative review from the commonality–individuality (C-I) perspective, a systematic literature search was conducted. We searched databases including Google Scholar and arXiv for publications between 2015 and 2025. The selection of these databases aimed to balance comprehensiveness with the inclusion of cutting-edge preprints common in this fast-moving field.

The search utilized a combination of core keywords to cover the relevant topics: “EEG” and “emotion recognition” as core domain; “deep learning” and its representative models (e.g., “convolutional neural network”, “recurrent neural network”, “graph neural network”, “transformer”) as core methods; “attention mechanism”, “domain adaptation”, “domain generalization”, “transfer learning” as additional key techniques. This keyword structure was designed to systematically capture studies at the intersection of the target physiological signal, computational task, and methodological paradigm.

The initial search returned approximately 18,900 records. Furthermore, citation tracking (snowballing) was performed on key articles to identify additional relevant studies.

### 2.2. Screening Process and Criteria

To ensure objectivity and reproducibility, a structured screening process was implemented. This process consisted of two sequential screening stages, each governed by explicit criteria.

#### 2.2.1. Initial Screening (Title/Abstract)

Records were excluded if they were clearly outside the scope, such as studies not focusing on EEG-based emotion recognition or not employing deep learning methods. This step resulted in approximately 260 articles proceeding to full-text review.

#### 2.2.2. Full-Text Assessment

The remaining articles were evaluated against pre-defined criteria:

The inclusion criteria included: (1) Research on EEG-based emotion recognition; (2) Core methodology based on deep learning; (3) Published in peer-reviewed journals/conferences or on recognized preprint servers (e.g., arXiv); (4) Full text available with complete experimental design and results.

The exclusion criteria included: (1) Non-English publications; (2) Short abstracts or papers with insufficient methodological detail; (3) Topic misaligned with the review’s focus; (4) Multimodal studies where EEG was a minor auxiliary signal; (5) Survey or review articles that did not introduce novel analytical frameworks.

These criteria ensured that the selected literature was not only thematically relevant but also methodologically sound and substantive enough for in-depth analysis.

#### 2.2.3. Final Inclusion

Following the full-text assessment, 76 studies were selected as the core corpus for in-depth analysis in this review. The final selection prioritized studies that were representative in terms of model architecture and performance, and particularly those whose findings or discussions informed the central theme of balancing commonality and individuality in EEG-based emotion recognition. All works discussed in the main text and presented in tables are drawn from this final set.

## 3. Background Knowledge of EEG Emotion Recognition

### 3.1. Emotional Models

Emotion is a subjective experience and corresponding behavioral response of an individual towards objective external events, reflecting the relationship between the subject’s needs and the objective external events. It is a mechanism gradually formed by humans in the process of adapting to the social environment. In 1997, Professor Picard from the Massachusetts Institute of Technology clearly defined the concept of affective computing in her monograph Affective Computing [1]: Affective computing refers to the computation of factors that are caused by emotions, related to emotions, or can influence and determine changes in emotions. Emotion recognition is a component of affective computing, focusing on how machines can accurately identify human emotional states. In the task of emotion recognition, it is necessary to establish a quantitative emotion model for computers to recognize emotions. Currently, researchers generally divide emotion models into two types: discrete models and continuous models.

(1) Discrete models

In 1971, Ekman [2] proposed a six-category emotion model: happiness, sadness, anger, fear, disgust, and surprise. In 1993, Lazarus [3] proposed a classification model containing 15 emotions. The vast majority of discrete emotion datasets directly use emotion categories as classification labels (such as happiness, sadness, anger, etc.), and the models are trained as multi-classification problems.

(2) Continuous models

**Figure 1:**
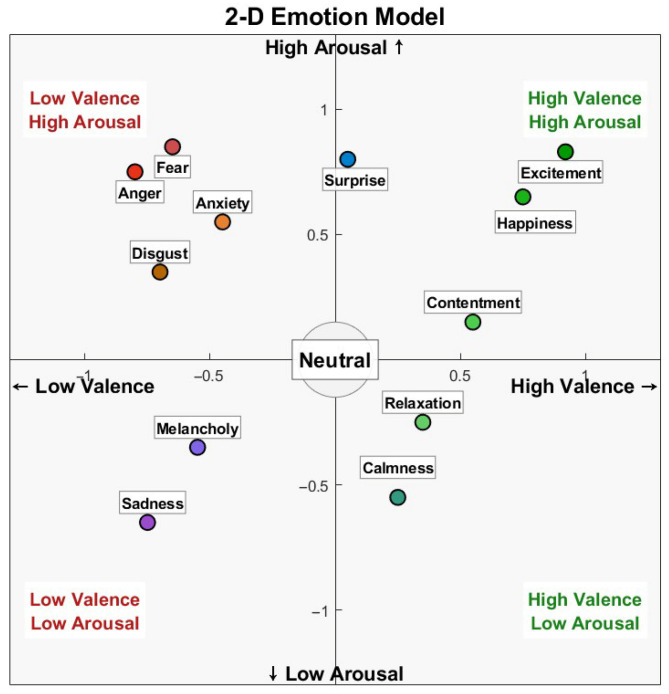
Two-dimensional Emotion Model.

In 1980, Russell [4] proposed the Two-Dimensional Emotion Model, which describes emotions using two basic dimensions: Valence and Arousal. Valence represents the change in emotion from negative to positive, while Arousal represents the change in emotion from low to high arousal. In addition, there are also three-dimensional emotion models based on Pleasure, Arousal, and Dominance. In the continuous model, various discrete emotional states can find corresponding dimensional combinations (as shown in Figure 1) Many emotion recognition datasets are constructed based on the two-dimensional continuous model.

### 3.2. Background of EEG

EEG is a technique that records the spontaneous electrical activity of the brain by placing electrodes on the scalp. The EEG signals reflect the synchronized electrical activity of large populations of neurons in the brain, and have high temporal resolution, which enables real-time monitoring of dynamic changes in brain activity. The close relationship between emotions and the brain is supported by numerous studies. From a neurophysiological perspective, emotional experiences are primarily based on the brain’s mapping and representation of bodily responses from the viscera, glands, and skeletal muscles in areas such as the brainstem, insula, and somatosensory cortex. These responses are ultimately processed in brain regions including the ventromedial prefrontal cortex, cingulate cortex, amygdala, and hypothalamus, which contribute to the main aspects of emotional experience. Therefore, in affective computing, using EEG and the interactions among multi-frequency neural oscillations to observe brain activity during emotional experiences is feasible for emotion recognition [5]. EEG signals, being temporal signals, can reflect the time-frequency characteristics of brain activity over time. Moreover, EEG is typically recorded from multiple channels. When electrodes are placed according to the 10–20 or 10–10 international electrode placement systems, the electrode locations correspond spatially to different brain regions. This allows EEG signals to capture spatial information about the activity in different brain regions and the interactions between them during emotional experiences.

### 3.3. Datasets

In recent years, with the increasing popularity of affective computing, many researchers have conducted relevant experiments and released several publicly available datasets based on physiological signals such as EEG. Among them, the SEED (SJTU Emotion EEG Dataset) and DEAP (Database for Emotion Analysis using Physiological Signals) datasets are the two most widely used datasets in affective computing based on physiological signals.

The SEED [6,7] is an emotion recognition dataset based on 62-channel EEG signals, which was publicly released by Professor Bao-Liang Lu from Shanghai Jiao Tong University in October 2015. The dataset currently consists of three subsets: SEED, SEED-IV, and SEED-VIG. SEED is the earliest released dataset with three emotion categories, SEED-IV is a dataset with four emotion categories, and SEED-VIG is a vigilance estimation dataset. The SEED dataset, which induces different emotions in subjects through movie video clips, mainly consists of two parts. One part is the EEG signals obtained during the experiment, which have undergone down-sampling, filtering, and artifact removal. The other part of the data involves feature extraction from the pre-processed EEG signals, including power spectral density, differential entropy, asymmetry difference in differential entropy, and asymmetry entropy of differential entropy, among other features. The features have also been smoothed using moving average and linear dynamic systems.

The DEAP [8] is a multimodal physiological database for affective computing, collected and made publicly available by Koelstra et al. from Queen Mary University of London. The sampled data includes 40 channels: 32-channel EEG signals, with the remaining channels comprising other physiological signals. In this database, participants’ emotions are elicited through music videos. During the experiment, participants watch 40 one-minute music videos and fill out the Self-Assessment Manikins (SAM). The SAM includes information on Arousal, Valence, Dominance, and Liking. The database also contains facial expression data for the first 22 participants while they were watching the videos.

Several studies have utilized other publicly available datasets such as DREAMER [9], MAHNOB-HCI [10], AMIGOS [11], and ASCERTAIN [12]. Developing open-source EEG datasets to evaluate recognition model performance remains an urgent and worthwhile research direction in this field. These datasets provide researchers with a shared benchmark, enabling fair comparisons and validation of results across different studies [13].

## 4. Overview of Deep Learning Framework

### 4.1. Deep Learning Models

Traditional emotion recognition methods rely on manual feature extraction and simple machine learning models, which have limitations when dealing with complex EEG signals. In recent years, with the rapid development of deep learning models, their application in EEG-based emotion recognition has become a research hotspot. Commonly used deep learning methods in the field of EEG emotion recognition include RNNs (Recurrent Neural Networks), CNNs (Convolutional Neural Networks), and GNNs (Graph Neural Networks). Among the deep neural networks applied to EEG signal processing, CNNs, RNNs, and DBNs (deep belief networks) perform better than other deep networks such as SAE (stacked autoencoders) and MLPNN (multilayer perceptron neural networks) [14].

RNNs are artificial neural networks designed for processing sequential data. Unlike traditional neural networks, RNNs have memory capabilities due to their hidden layers having recurrent connections. The output of a neuron is not only passed to the next neuron but also fed back to itself. This allows RNNs to capture temporal dependencies in sequences. The same parameters are used across different time steps, reducing the number of parameters to learn and enabling uniform processing of different positions in a sequence.

CNNs are deep neural networks designed for processing grid-structured data, such as images and audio, and have achieved significant results in the field of computer vision. The core component of a CNN is the convolutional layer, which performs convolution operations using convolutional kernels that slide over the input data to extract features. A single convolutional layer can contain multiple convolutional kernels to extract different features. The activation layer follows the convolutional layer, applying nonlinear transformations to the linear outputs using functions like ReLU, Sigmoid, or Tanh, enabling the learning of complex feature representations. The pooling layer performs down-sampling on feature maps to reduce data volume and computational complexity while preserving important feature information. Common pooling methods include max pooling and average pooling. The fully connected layer, located in the final layers of a CNN, integrates the features extracted by the convolutional and pooling layers to perform classification and output predictions. CNNs have strong local perception capabilities. The convolution operation allows them to learn local features, and the weight-sharing mechanism in convolutional layers reduces the number of parameters, lowering model complexity and enhancing generalization ability. Multiple convolutional layers, pooling layers, and convolutional kernels can be designed to extract high-level features.

GNNs are deep learning models designed for processing graph-structured data, where the input is a graph composed of nodes and edges. They learn node representations by aggregating neighborhood information. The core mechanisms are message passing and node updating, which involve message aggregation (a node collects information from its neighbors to generate an aggregated message) and node state updating (the node’s hidden state is updated using the aggregated message and its current state). A common variant is the GCN (Graph Convolutional Network), which defines graph convolutions and can be based on spectral or spatial methods. Spectral graph convolutions are based on spectral graph theory but have high computational complexity, especially with a large number of nodes. Chebyshev polynomial approximation methods can improve computational efficiency. Spatial graph convolutions operate directly on the graph’s spatial structure, mimicking the sliding window of image convolutions, and aggregate neighborhood information to update node features.

Deep learning models leverage hierarchical feature extraction to uncover deeper patterns from raw signals or manually extracted features. RNNs excel at capturing temporal dependencies in time series, particularly effective for processing emotional information that evolves over time in EEG-based emotion recognition. CNNs employ multi-layered convolutional and pooling operations to efficiently capture local features and spatial hierarchies, thereby extracting spatiotemporal characteristics. GNNs comprehensively understand the role of brain networks in emotions by aggregating and updating node features across graphs, revealing collaborative patterns between brain regions and their emotional associations. While shallow features reflect common patterns in underlying emotional EEG signals, deep features capture subtle and unique elements within training datasets, enabling the model to learn personalized characteristics. The discriminative power of deep learning models stems from their integrated grasp of both shallow and deep features. Shallow features identify universal patterns in EEG signals that mirror basic physiological mechanisms of emotion, such as generalized activation patterns and frequency variations across emotional states. These common patterns provide the model with general understanding of emotional categories. Meanwhile, deep features focus on uncovering unique individual traits in training data, often related to physiological differences, emotional expression styles, and specific situational responses. Through layered feature extraction, deep learning models strike a balance between universality and specificity, significantly enhancing their ability to distinguish different emotional states.

### 4.2. Performance Enhancement Modules and Strategies

In addition to the selection of model types, researchers also combine strategies to optimize network structure or improve classification accuracy, learning efficiency and generalization ability, such as attention mechanism, transfer learning, contrastive learning, etc.

The attention mechanism is one of the strategies that has been widely adopted by many researchers in recent years to enhance the performance of models. It is a type of dynamic weighting strategy in deep learning. In the early stages of the development of deep learning, Recurrent Neural Networks (RNNs) and Long Short-Term Memory networks (LSTMs) were the mainstream models for processing sequential data, such as in translation tasks. However, RNNs suffer from the problem of long-distance dependencies and are prone to forgetting early information when dealing with long sequence inputs. In 2014, Bahdanau et al. [15] first introduced the attention mechanism in their paper titled Neural Machine Translation by Jointly Learning to Align and Translate. The core idea was to enable the model to focus not only on the last hidden state but also dynamically attend to different parts of the input sentence and assign weights accordingly. With the attention mechanism, the model can flexibly determine which words are more important. In 2017, the Google team proposed the Transformer architecture in their paper Attention Is All You Need [16], completely abandoning RNNs or CNNs and instead using self-attention mechanisms to process the entire input sequence directly. The model computes attention weights by generating Q (Query), K (Key), and V (Value) matrices through linear transformations, allowing it to focus on the most relevant parts when processing sequences.

The attention mechanism is not a specific model in itself, but rather a strategy with various implementations. Its essence lies in assigning different weights to locate critical information and suppress irrelevant information, with the results presented in the form of a probability map or a feature vector. In models based on EEG signal analysis, there are three types of attention mechanisms: channel attention, temporal attention, and frequency-band attention. These mechanisms dynamically weight key information from spatial, temporal, and frequency dimensions, respectively, and have become the mainstream technological route for enhancing the performance of tasks such as emotion recognition and attention assessment. The subsequent sections will discuss separately the cases where the attention mechanism is used as a model improvement strategy and where it is employed as the core of a model.

Transfer learning is a machine learning approach that applies knowledge and feature representations learned in one or more source domains to target domains, thereby enhancing model performance in the target domain. This method proves particularly valuable when dealing with limited data or high annotation costs in the target domain. The core of transfer learning lies in leveraging similarities between source and target domains, transferring useful information from the source domain to the target domain. This approach reduces the required training data in the target domain while improving the model’s generalization capabilities. In deep learning-based EEG emotion recognition, integrating transfer learning with deep learning enables cross-subject and cross-dataset emotion recognition along with feature enhancement, effectively addressing three key challenges: individual differences, cross-dataset generalization, and data scarcity.

Moreover, the EEG emotion recognition methods based on single deep learning models such as RNNs, CNNs, and GNNs, as mentioned earlier, each have their unique advantages. However, it is challenging to represent all the information in emotional activities through a single model. Some studies have employed hybrid models, which combine multiple types of models in cascaded or parallel configurations to leverage the strengths of different models. In summary, the combination of hybrid models + attention mechanisms has become one of the most definitive and active technological approaches in the field of EEG emotion recognition over the past three years.

## 5. EEG Emotion Recognition Based on a Single Deep Learning Model

### 5.1. Emotion Recognition Based on RNNs

RNNs have a natural advantage in processing sequential data. The memory function of RNNs enables them to utilize past information to a certain extent in order to comprehend the current input. However, when dealing with long sequences, RNNs are prone to encountering the problems of vanishing or exploding gradients. One variant of RNNs, the Long Short-Term Memory network (LSTM), addresses these issues by introducing a gating mechanism to control the flow of information. This allows LSTMs to effectively capture long-term dependencies in sequences while preventing the gradual loss or unreasonable amplification of information as it propagates through the network. As a result, LSTMs are well-suited for processing EEG time-series signals.

For example, Alhagry et al. [17] proposed an end-to-end EEG emotion recognition classifier based on LSTM, which directly inputs segmented time series and classifies the sequence outputs through a fully connected layer. On the DEAP dataset, the classification accuracy rates were 85.65% for arousal, 85.45% for valence, and 87.99% for liking. Similarly to time-series data, one-dimensional features containing time-frequency information can also be fed into LSTM. Sakalle et al. [18] processed the data using Empirical Mode Decomposition (EMD) to extract features from multiple channels and different frequency bands, which were then input into LSTM. The model achieved an accuracy rate of 91.38% on the DEAP dataset and 89.34% on the SEED dataset using ten-fold cross-validation. Although sequence-to-sequence models can accomplish the task of emotion recognition, they fail to utilize the rich spatial information in EEG signals. Zhang et al. [19] introduced a novel deep learning framework called the Spatio-Temporal Recurrent Neural Network (STRNN), which captures spatial and temporal dependencies and integrates them into a unified spatio-temporal dependency model. The model also applies sparse projection to the spatio-temporal hidden states to identify salient regions with stronger discriminative power for emotion recognition. The model achieved an accuracy rate of 89.50% ± 7.63% on the SEED dataset.

In addition to using data from different frequency bands as separate features, Panachakel et al. [20] proposed training independent weights for each frequency band and then integrating the model predictions using a Majority Voting Classifier (MVC). Moreover, the model employed Common Spatial Patterns (CSP) to extract discriminative feature sets, thereby reducing the redundancy of information brought by multiple channels. The classification accuracy for all subjects exceeded 90%, with an average accuracy of 97.34% across all participants.

Beyond the conventional LSTM, some studies have introduced the Bidirectional Long Short-Term Memory network (Bi-Directional Long Short-Term Memory, Bi-LSTM) to achieve a balance between the range of receptive fields and the resolution of time series. Algarni et al. [21] proposed a model that extracts statistical features, wavelet features, and Hurst exponents, and employs a Binary Gray Wolf Optimization algorithm for feature selection. In the classification stage, a stacked Bi-LSTM is used. On the DEAP dataset, the model achieved average accuracy rates of 99.45% for valence, 96.87% for arousal, and 99.68% for liking.

RNNs are suitable for the direct input of time-series signals such as EEG and can extract information from a segment of the signal over time. However, they cannot directly represent the spatial information in EEG, which is crucial for emotion classification tasks, as spatial information from neural activities at different locations is highly relevant to emotional activities. In recent years, RNNs have increasingly been combined with other models to leverage their complementary strengths.

### 5.2. Emotion Recognition Based on CNNs

The basic input for CNNs is a two-dimensional matrix. EEG raw data are multi-channel time-series signals, which can be structured as a two-dimensional matrix with channels and time. In 2017, Cheng et al. [22] converted the DEAP dataset into a two-dimensional image format and directly input it into a CNN. Kwon et al. [23] utilized spectral features based on wavelet transforms and fed them into a CNN. Yang et al. [24] proposed a brain signal emotion recognition model based on a Multi-Column Convolutional Neural Network (Multi-Column CNN). By sampling at multiple time points and feeding them into multiple columns of convolution as inputs for independent recognition modules, the final decision was generated by the weighted sum of the outputs from each independent recognition module. On the DEAP dataset, the model achieved accuracy rates of 90.01% for valence and 90.65% for arousal.

The aforementioned two-dimensional matrix design primarily contains temporal information, and the processing of other time-domain and frequency-domain features follows a similar approach. Maheshwari et al. [25] extracted rhythmic features from each channel signal and fed them into a specially designed deep CNN. Additionally, there are methods that store time-frequency features in the structure of a two-dimensional time-frequency matrix. Wang et al. [26] proposed an EEG emotion recognition method based on Short-Time Fourier Transform (STFT) time-frequency features. Drawing on research regarding the relationship between emotional states and specific frequency bands and brain regions [27], the method selects symmetrical electrodes from the corresponding areas, fuses the STFT time-frequency features using multi-channel weighting, and inputs them into a CNN. This approach generates a fused EEG for each subject, thereby leveraging spatial information to some extent. The average classification accuracy on the DEAP dataset reached 82.88%.

Studies [28,29,30] have shown that emotions are related to the spatial information of brain activity. Taking this into consideration, Wen et al. [31] proposed a method for emotion recognition using CNN by converting the data into a two-dimensional matrix based on the Pearson correlation coefficient matrix. The study explored different transformation algorithms to ensure the maximization of the Pearson correlation coefficient information of adjacent information. The results showed that the method of channel reordering based on the algorithm for maximizing adjacent information achieved accuracy rates of 77.98% for valence and 72.98% for arousal on the DEAP dataset. Similarly, Moon et al. [32] also considered the issue of adjacency matrix channel ordering. Based on the physical locations of the electrodes, the study employed a spiral ordering that ranged from fewer interhemispheric crossings to more interhemispheric crossings. It was demonstrated that incorporating asymmetric information within the receptive field of the CNN can enhance model performance.

To represent spatial information corresponding to physiological structures within CNNs, Liao et al. [33] transformed multi-channel time series into two-dimensional matrices, preserving the positional relationships and symmetry information of the channels. Based on a similar spatial encoding approach, Cui et al. [34] proposed an end-to-end Region Asymmetric Convolutional Neural Network (RACNN) that simultaneously learns regional information between adjacent channels and asymmetry features between the two hemispheres of the brain. This model achieved average accuracy rates of 96.65% for valence and 97.11% for arousal on the DEAP dataset, and 95.55% for valence and 97.01% for arousal on the DREAMER dataset. Li et al. [35], through spatial encoding and frequency-decomposed features, constructed a Spatial-Temporal-Connective Multi-Scale Convolutional Neural Network (STC-CNN). This network captures the synergistic mechanisms between brain regions under emotional stimuli by computing inter-channel connectivity. It achieved average accuracy rates of 96.79% for valence and 96.89% for arousal on the DEAP dataset. Ding et al. [36] introduced the TSception multi-scale convolutional neural network, which captures temporal dynamics and spatial asymmetry through multi-scale spatio-temporal convolutional kernels. Additionally, a higher-order fusion layer was designed to optimize the network structure by learning global representations of the hemispheres. Under the same settings, this network outperformed benchmark methods such as EEGNet [37], SCN (ShallowConvNet) [38], and DCN (Deep ConvNet) [38] on both the DEAP and MAHNOB-HCI datasets in most cases, while also having a smaller number of parameters. Interpretability studies have shown that the network indeed learns information from the correct regions, providing a foundation for the development of subsequent models.

In addition to traditional CNNs, Capsule Networks [39] (CapsNet) have also been applied to EEG emotion recognition tasks as a variant of CNNs. Liu et al. [40] proposed the Multi-Level Features Guided Capsule Network (MLF-CapsNet), which constructs primary capsules by integrating multi-level feature maps learned from different layers, thereby enhancing feature representation capabilities. Li et al. [41] proposed the MTCA-CapsNet model, which is based on multi-task learning with capsule networks and attention mechanisms. This model achieves simultaneous learning by exploring the commonalities and differences between tasks and acquires more data from different tasks to improve generalization and robustness. On the DEAP dataset, the average accuracy rates for arousal, valence, and dominance were 97.41%, 97.24%, and 98.35%, respectively. On the DREAMER dataset, the corresponding accuracy rates were 95.54%, 94.96%, and 95.52%. Addressing the limitations of existing shallow convolutional neural networks in effectively representing spatial relationships between different features and the issue of limited sample sizes, Fan et al. [42] proposed the Light-weight Residual Convolution-based Capsule Network (LResCapsule). This network extracts spatial features and local patterns at different scales and frequencies through a light-weight network structure, learning relationships between features and hierarchical features. On the DEAP dataset, the accuracy rates for arousal, valence, and dominance were 97.58%, 97.45%, and 97.61%, respectively. On the DREAMER dataset, the accuracy rates reached 95.77%, 95.15%, and 95.59%. To address the cross-subject task, Liu et al. [43] constructed the Domain Adaptation-based Multi-branch Capsule Network (DA-CapsNet), which embeds adversarial domain adaptation into the capsule network to reduce the distribution gap between the source and target domains, thereby improving cross-subject accuracy. This model has shown good performance on the DREAMER, DEAP, and SEED datasets.

While existing extensive research has been conducted on CNN emotion recognition models, several challenges remain: Direct input of two-dimensional matrices results in loss of spatial information; spatial encoding, while capturing spatial features, typically employs compact representations that fail to directly capture dynamic patterns; and deep CNN architectures demonstrate high computational resource consumption. These limitations indicate the need for optimization in both feature engineering and model architecture for single CNN models.

### 5.3. Emotion Recognition Based on GNNs

The concept and computation of brain networks have evolved from the notion and calculation of brain connectivity. When researchers realized that connectivity alone was insufficient to explain cognition and disease, they introduced graph theory, abstracting connections into the form of nodes, edges, and graphs. This transition elevated the concept to the system-level idea of brain networks. Graph theory metrics are then employed to quantify global or local characteristics of the brain network, such as the small-world index, global efficiency, node degree, clustering coefficient, characteristic path length, etc. The brain’s functional network is an abstract construct. Each electrode region we define as a measurement area corresponds to a node in the network. By extending this concept along the temporal dimension, we can construct dynamic brain networks. Additionally, we can repeatedly build networks for the same set of brain regions across multiple dimensions, modalities, frequency bands, and time points. Interactions across these layers can then be described using intra-layer and inter-layer connections, thereby constructing multilayer brain networks.

Wang et al. [44] proposed the P-GCNN (Phase-Locking Value Based Graph Convolutional Neural Networks) model, a method for emotion recognition based on graph convolutional neural networks using Phase-Locking Value (PLV). This approach models univariate EEG signal features as multivariate graph signal features based on a brain network structure constructed using PLV. It achieved a classification accuracy of 84.35% on the SEED dataset and average accuracy rates of 73.31% for valence, 77.03% for arousal, and 79.20% for dominance on the DEAP dataset. Zhang et al. [45] introduced the Graph Convolutional Broad Network (GCB-Net), which combines graph convolution with broad learning. To extract dynamic, multilayer, and adaptive graph features, Song et al. [46] proposed the Dynamic Graph Convolutional Neural Network (DGCNN) in 2018. This model can simultaneously optimize the graph neural network model (weights) and the single-layer graph structure. It adaptively learns the intrinsic associations between EEG channels by optimizing the adjacency matrix, using the Backpropagation (BP) algorithm to optimize the adjacency matrix until an optimal or suboptimal solution is obtained. In 2023, Jang et al. [47] and Moon proposed an end-to-end adaptive graph neural network approach that jointly learns sample-specific multilayer connectivity structures and signal features directly from raw EEG data, without requiring prior knowledge of graph structure and features. The model achieved an accuracy of 73.5% ± 8.07% on the DEAP dataset and 55.5% ± 7.59% on the DREAMER dataset.

According to prior research [48], incorporating sparsity in the topology of EEG channels can alleviate the over-smoothing issue, preserve key functional and structural information, eliminate redundant connections, support large-scale network analysis, and enhance the model’s robustness to noise and individual differences, thereby more accurately reflecting the functional organization of the brain. Zhong et al. [49] proposed the Regularized Graph Convolutional Neural Network (RGCNN), which captures both local and global associations among different EEG channels by considering the biological topological structure between brain regions. Analysis also revealed that the model identified meaningful brain regions and inter-channel relationships.

In recent years, several studies based on Graph Neural Networks (GNNs) have also incorporated attention mechanisms. Li et al. [50] proposed a method called the EEG Correlation Analysis-guided Graph Local Enhanced Feature Learning Network (CAGLE-net). This method captures emotional topology using static connectivity based on Pearson Correlation Coefficient (PCC) and Spearman’s rank Correlation Coefficient (SCC). The brain is divided into five physiological regions: frontal, temporal, central, parietal, and occipital. Local features are read out in parallel through Graph-attention Readout (GARO) and Squeeze-Excitation Readout (SERO), and then aggregated to generate discriminative representations through a Multi-Head Cross-Attention Fusion Layer (LEFL). This approach achieved an accuracy rate of 95.03% ± 3.76% on the SEED dataset. Fu et al. [51] introduced a Dynamic Graph Attention Network (DGAT), which constructs dynamic graphs that change over time and encodes features through a dual-branch encoder with spatial branches and channel-frequency attention. The features from the two branches are concatenated and passed through a fully connected layer to output classification labels. Jiang et al. [52] proposed Temporal–Spatial Attention Neural Networks with Task-Specific Graph (TSANN-TG), which synchronously extracts EEG time-frequency features using temporal-spatial attention to generate attention maps that reveal nonlinear neural relationships. The method further introduces task-specific graphs that adaptively reconstruct graph structures based on individual brain region connectivity patterns, achieving unique topologies for different emotional categories. Experiments on a self-built dataset and the DEAP dataset, along with comparative and ablation studies, validated the model’s performance. Liu et al. [53] proposed a Dynamic Graph Attention Network with Multibranch Feature Extraction and Stage Fusion (MASDGAT-Net). This network designs modular feature extraction and fusion branches, efficiently extracting key channel-frequency information from EEG features through attention mechanisms. It also incorporates a broad learning system to further explore brain connectivity feature information.

In addition, many studies have focused on alleviating inter-subject differences in EEG feature distributions but have overlooked the dynamic EEG connectivity patterns and prediction biases caused by individual differences. This oversight may lead to poor generalization of models to unseen subjects. Chen et al. [54] proposed a Graph Domain Disentanglement Network (GDDN), which extracts commonalities and specificities in EEG graph connectivity and graph representations through a graph domain disentanglement module, thereby enhancing the network’s generalization ability to unseen individuals. Experiments using the SEED, SEED-IV, and MPED datasets demonstrated that GDDN achieved superior performance in cross-individual emotion recognition tasks and also investigated the performance after cross-dataset transfer.

In recent years, brain network features and Graph Neural Networks (GNNs) have been used to reflect information exchange between different brain regions. Before features enter the GNN, graph features are designed and combined with performance-enhancing modules such as multi-task learning, transfer learning, and attention mechanisms within the network. This approach has effectively improved emotion recognition performance and generalization ability.

## 6. EEG Emotion Recognition Based on Deep Learning Hybrid Model

In recent years, hybrid models based on deep neural networks have been widely applied in the field of EEG-based emotion recognition. These models significantly enhance the performance of emotion recognition by integrating different types of neural network architectures.

In 2016, Li et al. [55] proposed the CNN-LSTM method, which enables the model to utilize spatial information and time-related information between frame sequences. Kang et al. [56] introduced a data augmentation and ensemble deep network based on ICA-Evolution. After removing artifacts through ICA decomposition, synthetic samples are generated by evolving and perturbing the source components, thereby alleviating the issue of insufficient data. This network has CNNs on both the temporal and frequency axes, integrating information from both axes before inputting it into LSTM. The model achieved an accuracy rate of 80.99% ± 6.47% on the DEAP dataset. In 2018, Yang et al. [57] proposed a Parallel Convolutional Recurrent Neural Network (PCRNN). After spatial encoding of multi-channel time series, parallel CNN and RNN branches are used to extract spatial features (local associations between electrodes) and temporal dynamics (such as the gradual process of changing emotional states), respectively. On the DEAP dataset, the model achieved accuracy rates of 90.80% ± 3.08% for valence classification and 91.03% ± 2.99% for arousal classification. Furthermore, some hybrid models have considered incorporating multiple spatiotemporal information sources. However, the extraction processes of temporal information and spatial features are often disconnected, potentially leading to the loss of some spatial features. To address this, Guo et al. [58] constructed a Convolutional Gated Recurrent Unit-driven Multidimensional Dynamic Graph Network (CGRU-MDGN). This net-work replaces the fully connected layer of the GRU with a convolutional layer to capture both local spatial features and temporal information. It generates various non-Euclidean space representations through multidimensional dynamic graph convolutions and then fuses features from different dimensions or perspectives using an adaptive weighted sum.

In addition, Song et al. [59] proposed the Instance-Adaptive Graph (IAG) method in 2020, which automatically acquires sample-specific multilayer graph structures by manually extracting features, presenting variable graph representations determined by different input instances. An additional branch is introduced to characterize the intrinsic dynamic associations between different EEG channels. By designing multilevel multigraph convolution operations and graph coarsening mechanisms, more accurate graph representations are achieved. The dependencies between regions after sparsification are established through LSTM to extract more discriminative feature vectors. Experiments on the SEED dataset showed an accuracy rate of 95.44% ± 5.48% in subject-dependent experiments and 86.30% ± 6.91% in subject-independent experiments. In 2023, Song further proposed the Variational Instance-Adaptive Graph [60] (V-IAG), which introduces a variational branch on the basis of IAG to estimate potential uncertainty information. The variational branch generates probabilistic graphs to quantify uncertainty. Moreover, Liu et al. [61] proposed a network framework combining Sparse graphic attention LSTM (SGA-LSTM). This framework, through its dual-branch structure design, extracts global features that reflect the intrinsic associations between different channels by adding attention to specific channels, effectively enhancing the role of channel information closely related to emotional fluctuations while weakening the impact of channels with weaker responses. Additionally, although convolutional kernels in CNNs can perceive local features, they may disrupt the temporal context existing between frames. To address this, Gong et al. [62] proposed the Attention-based Convolutional Transformer Neural Network (ACTNN), which integrates spatial, spectral, and temporal information by cascading convolutional neural networks and Transformers. The model uses CNNs to extract local spectral-spatial features and employs a multi-head attention mechanism to achieve global feature perception. The average recognition accuracy rates on the SEED and SEED-IV datasets reached 98.47% and 91.90%, respectively.

In recent years, there have also been studies that combine CNNs or GNNs with RNNs to process spatial and temporal information separately and then enhance classification performance with attention mechanisms. In 2024, Houssein et al. [63] proposed the TFCNN-BiGRU architecture, which uses TFCNN (Time-Frequency Convolutional Neural Network) integrated with time-frequency features and Bi-GRU (Bidirectional Gated Recurrent Unit) to extract temporal features and local spatial features, and improves recognition accuracy with the help of self-attention mechanisms. In the same year, Tang et al. [64] proposed a framework based on Multi-Domain Based Dynamic Graph Representation Learning (MD^2^GRL), which uses GRU and PSD to extract time-domain and frequency-domain features from EEG signals. It introduces self-attention mechanisms and a soft threshold operator to construct a sparse adjacency matrix for dynamic graph representation. It then uses a dual-branch GNN module to capture the topological relationships and rich features within the graph. Additionally, Liu et al. [65] proposed ERTNet, an interpretable end-to-end EEG emotion recognition framework based on a hybrid CNN and Transformer architecture. It achieved an accuracy rate of 74.23% ± 2.59% on the DEAP dataset and 67.17% ± 1.70% on the SEED-V dataset. Through interpretability analysis, it was confirmed that the beta and gamma frequency bands have the most significant impact on emotion recognition performance.

Focusing on both inter-subject differences and commonalities in tasks can effectively enhance model performance. Zhang et al. [66] proposed the Commonality and Individuality-based EEG Graph learning network (CI-Graph), which designs a commonality–individuality dual-branch structure: The C-Graph uses Graph Convolutional Networks (GCN) to extract shared emotional representations across subjects, while the I-Graph employs a Tokenized Graph Transformer (TokenGT) and graph diffusion convolution to capture individual-specific features. The Graph Learning (GL) module dynamically optimizes the adjacency matrices of both graphs. By jointly performing multi-task classification, regression, and contrastive learning, the model significantly improves generalization across subjects.

The hybrid models introduced above all enhance feature extraction, classification, and generalization performance by integrating the structures and functions of individual models into modules or branches to form more complex model architectures. These hybrid models have advantages in comprehensively utilizing various types of information in EEG to improve classification accuracy and can be studied from multiple angles regarding model interpretability. However, hybrid models often suffer from complex structures and a large number of parameters, making real-time online classification applications challenging. Optimizing and balancing the trade-offs between classification performance, generalization ability, and parameter quantity in models may be a promising research direction.

## 7. Other Performance Improving Methods

### 7.1. EEG Emotion Recognition with Attention Mechanism

Introducing attention mechanisms enables models to focus on brain regions and interactions most relevant to emotional states, allowing them to prioritize discriminative features. Two implementation approaches exist: one leverages existing deep learning models by incorporating additional attention modules that assign weights across different dimensions; the other centers on specialized attention architectures, such as transformer models for emotion recognition. In recent years, numerous models have incorporated attention modules into CNNs, RNNs, or hybrid architectures, applying temporal, spatial, spectral, and graph-based attention weights to diverse features.

For instance, Tao et al. [67] proposed ACRNN, which adaptively allocates weights to different channels and utilizes convolutional neural networks to extract spatial information from encoded EEG signals. To explore the temporal information in EEG signals, they integrated extended self-attention into RNNs to re-encode time-based intrinsic similarity importance within EEG signals. Hu et al. [68] employed concatenated self-attention modules by cascading Convolutional Neural Networks (CNNs) and Long Short-Term Memory Networks (LSTM). Liu et al. [69] utilized pre-trained capsule networks combined with coordinate attention to inject relative spatial information into input signals, then mapped EEG signals into higher-dimensional spaces. By embedding positional information into channel attention mechanisms, this approach enhanced feature representation capabilities and improved model performance. Arjun et al. [70] presented a CNN with attention framework to perform subject-independent emotion recognition on the encoded lower dimensional latent space representations obtained from the proposed LSTM with channel-attention autoencoder. With the attention mechanism, the proposed approach could highlight the significant time-segments of the EEG signal, which contributes to the emotion under consideration as validated by the results. Similarly, there is DACB [71], a hybrid deep learning model, which integrates dual attention mechanism, CNN and Bi-LSTM for effective emotion classification. In the CNN-based spatial feature extraction module, Ma et al. introduced a 1D-SE (One-Dimensional Squeeze-and-Excitation) attention mechanism to enable the model to learn the importance of various channel features. Meanwhile, the Dot Multiplier Attention Mechanism is applied in the feature selection module, which helps the model recognize the significance of both spatial and temporal features.

Transformer possesses global attention, which can capture long-range dependencies compared to CNN-based models. It maps different dimensional features of EEG signals (such as frequency domain, spatial domain, and temporal domain) into a unified feature space (semantic space), enabling simultaneous feature extraction and fusion across multiple dimensions including frequency domain, spatial domain, and temporal domain. Gong et al. introduced a novel attention mechanism that significantly enhances the spatial and spectral distinguishability of EEG signals, making the features input into the Transformer more discriminative. This approach leverages the transformer’s multi-head self-attention mechanism while integrating the localized perception capabilities of CNN with the global perception strengths of the transformer.

Examples above show the attention module can be applied to stages like feature extraction and feature selection to let the deep learning model focus on more discriminative features and representations, and can better extract the commonalities of emotion states.

### 7.2. EEG Emotion Recognition with Transfer Learning

In the field of EEG emotion recognition, different subjects, sessions and datasets can be treated as “domains” as long as they have significant distribution differences. In transfer learning, the common features of the underlying representation are often frozen, fine-tuned, or differential learning rates are adopted for the upper and lower levels to take advantage of the common features of the source domain and the individuality of the target domain. There are two main branches in transfer learning that can improve the adaptability and generalization ability of the model by combining commonness and individuality. One is Domain Adaptation, and the other is Domain Generalization.

DA methods increase the accuracy of the target data by minimizing domain shifts between source and target domains, indicating that during the training phase, we must have obtained the data from the target domain. Since 2016, DA methods have been introduced into EEG emotion recognition, parameter transfer, DANN (Domain-Adversarial Neural Network) and other methods to achieve transfer learning and learn domain invariant representation [72,73], DA methods can be combined with deep-learning based feature extraction. Guo et al. [74] constructs a novel multi-source domain adaptation with a Spatio-temporal feature extractor (MSDA-SFE) for EEG emotion recognition, which could reduce the signal differences between subjects and between sessions to make the target domain features align with the source domain features. The model uses Bi-LSTM and CNN to capture the sequence features and local spatial features of brain signals, respectively, and then estimates the difference between target domain features and each source domain features through the maximum mean difference (MMD) loss function to gradually align the feature distribution. The results of Cross-subject and Cross-session experiment were 91.65 ± 02.91% and 92.12 ± 07.12%, respectively. Imtiaz et al. proposed GPTDS (Gradual Proximity-guided Target Data Selection), to mitigate negative transfer caused by diverse and unreliable samples. The method gradually selects reliable target domain samples for training by considering their proximity to source clusters and the model’s confidence in predicting them. They also introduced a cost-effective TTA (Test-Time Augmentation) technique which applies augmentation only when necessary, thereby improving model performance during inference and reducing computational costs.

On the other hand, DG methods learn across multiple source domains to enhance model generalization, enabling models to handle unseen domains, thus eliminating the need for target domain data. These approaches retain common emotional features while adapting to each domain’s unique characteristics, thereby improving the model’s performance in the target domain. Ma et al. [75] proposed an adversarial domain generalization framework DResNet (Domain Residual Network) to address individual differences in EEG data. By introducing domain generalization methods and improving deep adversarial networks for domain generalization, they developed the DG-DANN (Domain Generalization) method based on DANN. This approach connects feature extractors and domain classifiers through a gradient reversal layer, enabling the feature extractor to generate domain-invariant features during training. Sun et al. developed a deep learning approach that integrates domain adversarial transfer networks with attention mechanisms. The hierarchical architecture extracts multi-scale EEG features: the lower layers capture local dynamics, the middle layers extract rhythmic information, and the upper layers acquire contextual semantics. Individual attributes (age, gender, educational background) are encoded and aligned with EEG features for fusion. By combining channel attention and extended spatial attention in a dual attention module, the system adaptively fuses multi-scale features to enhance emotional discrimination capabilities.

However, DG methods without target domain data are not necessarily the most effective methods to achieve high cross-subject accuracy. In recent years, some studies have demonstrated that efficient cross-domain transfer learning can be carried out with a small amount of target domain data, and show certain potential for practical deployment [76,77].

### 7.3. Neural Inspired Methods

In addition to deep learning and its model performance improvement module, some studies have introduced methods inspired by psychology or neuroscience. Shen et al. [78] proposed a contrastive learning method for inter-subject alignment inspired by ISC (inter-subject correlation) studies. The ISC studies have focused on the inter-subject consistent neural activities in response to the same naturalistic stimuli or in the same social interaction scenarios. On the SEED dataset, it achieved a three-class classification accuracy of 86.4 ± 6.4%, better than the DResNet. By analyzing the similarity of EEG activity across subjects viewing identical videos, researchers can better identify commonality in emotional brain signals. In 2025, DAST [79] was proposed, which further applied contrastive learning framework on a temporal and spatial transition convolution (TSTC) module with a dynamic attention (DyA) module. The model also achieved state-of-the-art performance for cross-subject tasks. However, individual differences across domains remain a critical consideration. For instance, when exposed to the same video stimuli, participants may experience varying emotional responses due to differences in personal experiences and memories. All this information can be difficult to captured by traditional machine learning methods. Some studies have integrated psychological metrics such as the Big Five personality traits, which show correlations with emotional EEG patterns [80]. The EEG emotion recognition model combined with relevant psychological indicators can improve the accuracy of practical application through these clues.

## 8. Model Comparisons and Future Challenges

To comprehensively evaluate the performance of various deep learning models in EEG-based emotion recognition, this section selects and analyzes representative models that are innovative and aligned with the review’s structure, while considering citation counts and journal prestige. In all compared experiments, datasets were divided into training sets, validation sets, and test sets according to predefined ratios. Table 1 details the datasets utilized by these models, along with their accuracy evaluation metrics.

In addition to the display of mean accuracy and standard deviation, Table 1 illustrated effect size to better indicate the magnitude and practical significance of performance improvements beyond mere statistical differences. By employing ΔAcc (the absolute difference in accuracy), the table provides a direct, interpretable metric of how much a proposed model surpasses its baseline. The ΔAcc values provide an intuitive measure of improvement magnitude, enabling a quick overview of claimed advancements. Their adoption here addresses common reporting limitations: incomplete variability metrics (e.g., missing per-fold results, precluding standardized effect sizes like Hedges’ g), significant heterogeneity in validation paradigms and tasks, and the varying strength of baseline methods across studies.

However, direct comparison of ΔAcc values across all rows is not advised. This metric does not account for baseline difficulty (where large gains over weak baselines may be less meaningful), ignores variance critical for assessing robustness, and conflates improvements from tasks and datasets of fundamentally different complexity. Therefore, ΔAcc is most reliably interpreted within a single row-comparing a model to its specified baseline under identical conditions. While this table offers a valuable overview, it underscores the need for more uniform reporting to enable rigorous cross-study synthesis.

Despite the accuracy-oriented comparisons in Table 1, an early and persistent observation is that classification rates drop sharply when models are shifted from subject-dependent to subject-independent evaluation protocols. A parallel trend is evident in the ΔAcc values. The data reveals a clear hierarchy of improvements: deep learning models of various types achieve rapid and significant gains in effect size over traditional machine learning methods (e.g., SVM), demonstrating their fundamental advantage in feature representation. More recent studies, which use established deep learning models (e.g., DGCNN) as benchmarks, show continued yet more modest optimization. Within this progression, a critical pattern emerges: while specially designed approaches (e.g., incorporating attention, transfer leaning, contrastive learning) for subject-independent scenarios do outperform their generic deep learning counterparts, the magnitude of this improvement is considerably smaller than the initial leap from traditional to deep learning methods. This suggests that while architectural innovations are beneficial, they have not yet produced a transformative breakthrough in generalizability. This persistent and recalcitrant performance gap underscores a core limitation: despite increasingly complex architectures, stably overcoming individual differences remains a fundamental, unsolved challenge. Subsequent studies have attempted various remedies, yet no consensus metric exists to evaluate how well a method balances “working for everyone” versus “recognizing this particular person”. To fill this gap, we propose a lightweight C-I (commonality–individuality) assessment framework that scores each model on commonality gain, individuality preservation, and explicitness of their trade-off mechanism.

The rationale for these three dimensions is as follows:

(1) Hierarchical feature nature: Regardless of training protocol, deep networks inherently extract broad, subject-invariant patterns in shallow layers while encoding finer, subject-specific details in deeper layers.
(2) Subject-/stimulus-dependent boundary: The exact depth at which “common” ends and “individual” begins is not fixed; it shifts across subjects and stimuli. Treating this boundary as a tunable variable—for example via transferable layer selection in domain adaptation—allows the model to self-optimize for each target scenario.
(3) Real-world, few-shot demand: Under subject-independent or small-sample conditions that mimic real-life deployment, explicitly balancing commonality and individuality has repeatedly raised classification accuracy.

Such a lightweight C-I scorecard, as shown in Table 2, is only an example of applying this C-I framework to assess existing models for EEG-based emotion recognition. It can also be seen that the models in the table that better meet our three criteria also perform better in cross-subject studies.

Despite the widespread application of deep learning methods and their improvements in the field of EEG-based emotion recognition in recent years, there still remain several issues and challenges that merit further investigation.

(1) High-quality data acquisition research: The quality of EEG signals is sensitive to factors such as the experimental environment, subject and equipment conditions, and the operation of experimental personnel. For human subjects of different ages and genders in natural and open states, how to minimize irrelevant interference factors and ensure the unified acquisition of high-quality EEG signals requires more research on hardware and experimental settings [81]. Datasets with large number of subjects like THU-EP and FACED also show potentials in grasping commonalities across domains.
(2) Feature selection issues: The choice of deep learning models also imposes requirements on the data structure of input data or features. How to select appropriate features remains a research question. There are numerous ways to extract features from EEG signals, including power spectral density, differential entropy, asymmetry difference in differential entropy, asymmetry ratio of differential entropy, discrete wavelet analysis, empirical mode decomposition, and statistical features (mean, variance, etc.). How to extract suitable features or integrate different features will significantly impact affective computing models.
(3) Classification effect evaluation: To verify the model’s performance in various aspects and meet the needs of different evaluation criteria, multiple experimental strategies have been proposed successively. Different strategies vary in application scenarios and recognition difficulty. Therefore, different datasets have differences in experimental settings and label settings. Some emotion recognition studies use classification accuracy on public datasets to reflect model performance. However, due to differences in task settings (such as cross-subject or subject-dependent, validation methods, etc.), it is difficult to directly compare performance through accuracy mean and variance. In addition to the heterogeneity of the studies, the lack of detailed experimental data in each study report has also hindered attempts to compare model effects uniformly using methods such as model effect size. How to establish an effect evaluation that is less sensitive to different experimental settings awaits further research.
(4) Model interpretability research: In EEG-based deep learning emotion recognition, model interpretability is an important research direction. There are passive methods (interpreting the model after training is completed) and active methods (incorporating interpretability design during model training) in interpretability research. Model interpretability involves understanding the internal working mechanisms of the model and combining prior knowledge with data-driven methods to build and optimize deep models. This helps us understand model decisions, verify model reliability, and guide model design [82].
(5) New models and strategies: In addition to the application of models such as RNNs, CNNs, and GNNs, some new models (such as spiking neural networks) [83] combining the multi-domain EEG signatures [84] and gradually being tried in emotion recognition tasks. Similarly, performance enhancement strategies and modules that integrate attention mechanisms and transfer learning are increasingly being applied. For example, continuous representations of emotion EEG signals may provide better solutions to monitoring emotion changes overtime. Compared with the deep learning models currently used in emotion recognition research in affective computing, methods based on new models and strategies may perform better in adapting to small sample sizes, model parameters, cross-subject, cross-session, and cross-dataset aspects. Our proposed C-I framework could serve as a valuable tool in the exploration of these new models and strategies. It might help predict how the integration of psychological insights and feature innovations would perform in emotion recognition tasks.

## 9. Conclusions

This article reviews recent deep learning-based EEG emotion recognition methodologies, systematically analyzing research from both commonality and individuality perspectives. It examines different deep learning models for emotion identification and proposes strategies combining deep learning with transfer learning to enhance classification accuracy. The study begins by introducing fundamental theories of EEG-based emotion recognition, including commonly used datasets, deep learning models, performance-enhancing modules, and optimization techniques. Subsequently, it discusses the applications and developments of RNNs, CNNs, GNNs, and hybrid models, along with frameworks incorporating attention mechanisms, transfer learning, etc. Finally, the paper identifies existing challenges in current deep learning-based emotion recognition research and offers some observations for future improvements.

(1) Deep learning-based methods shows capability of capturing the features of emotion activity on different dimensions, a hybrid model is more capable of utilizing multi-dimensioned information. Combing attention mechanism helps the model to focus on more discriminative features and to reduce complexity. However, the drop in accuracy when shifted from subject-dependent to subject-independent training posed the challenge to deep learning-based methods.
(2) The feature discrimination of the model comes from the grasp of commonality and individuality of shallow and deep features. Deep learning methods themselves attain different levels of commonality and individuality, but is sensitive to changes in training methods. Strategies like transfer learning and contrastive learning can alleviate the dilemma of accuracy degradation under cross-subject or cross-domain experimental settings, thus improving generalization ability. This indicates that—whether or not they are explicitly stated—model architecture, training protocols, datasets and even experimental designs for emotion recognition must all take into account commonality, individuality, and the trade-off between them under specific methodological conditions (e.g., how many layers are frozen in transfer learning, or whether the experimental design emphasize the number of subjects versus the consistency of emotion elicitation).
(3) Everything, from features to representations, from machine pattern recognition tasks to the model’s interpretable common patterns of emotions revealed by EEG signals and prior knowledge, combined with the rapid adaptation of the individuality of a single human brain in the actual application scenario, points to a possible solution of integrating the feature-extracting ability of deep learning models with strategies like transfer learning and contrastive learning.

At last, we must point out several limitations of our work. Firstly, this article is positioned as a narrative review; it thematically integrates recent advances in deep-learning-based EEG emotion recognition from the commonality–individuality perspective rather than answering a single, narrowly defined research question. Consequently, the PRISMA flow-chart and checklist were not applied.

Secondly, the literature discovery relied mainly on Google Scholar using keyword bundles. Representative, highly cited and methodologically diverse studies were prioritized; additional references were identified by citation snowballing. While this iterative strategy captures the field’s mainstream trends, it may introduce selection bias and does not guarantee exhaustiveness or full reproducibility.

Additionally, Table 2 is intentionally small and qualitative. It covers only a handful of methods, uses discrete symbols rather than weighted scores, and does not constitute a full systematic assessment. Thus, it should be viewed as an illustrative snapshot of the Commonality–Individuality framework rather than an exhaustive ranking; expanding it into a large-scale, quantitative scorecard (with expert weighting or public voting), is left to future work.

## Figures and Tables

**Table 1:** The representative deep learning-based EEG emotion recognition studies.

Author	Year	DL Model	Datasets	Accuracy ± Standard Deviation *	Effect Size (ΔAcc)
Moon, et al. [32]	2018	CNN	DEAP	99.72% (**D**, best)	+44.30% (**D**; vs. SVM)
Zhang, et al. [19]	2019	STRNN	SEED	89.50% ± 7.63% (**S**)	+5.51% (**S**; vs. SVM)
Yang, et al. [24]	2019	Multi-Column CNN	DEAP	90.65%, 90.01% (Arousal, Valence)	+23.55%, +19.11% (**D**; Arousal, Valence; vs. SVM)
Song, et al. [46]	2020	DGCNN	SEED, DREAMER	subject-dependent: 90.40% ± 8.49% (**S**) 84.54% ± 10.18%, 86.23% ± 12.29%, 85.02% ± 10.25% (**DR**; Arousal, Valence, Dominance) subject-independent: 79.95% ± 9.02% (**S**)	subject-dependent: +6.41% (**S**; vs. SVM) +15.70%; +26.09%; +9.18% (**DR**; Arousal, Valence, Dominance) subject-independent: +16.31% (**S**; vs. TCA, Transfer Component Analysis)
Song, et al. [59]	2020	IAG	SEED, MPED	95.44% ± 5.48% (**S**; subject-dependent) 86.30% ± 6.91% (**S**; subject-independent) 8 types of combinations in form of positive-neutral-negative: 74.77% ± 10.75% 3 categories: 73.58% (**M**), 68.41% (F1 score) 7 categories: 40.38% ± 8.75% (**M**)	+11.45% (**S**; subject-dependent; vs. SVM) +22.66% (**S**; subject-independent; vs. TCA) 8 types of combinations in form of positive-neutral-negative: +14.91% (**M**; vs. SVM) 3 categories: +16.52% (**M**; vs. SVM) 7 categories: +9.24% (**M**; vs. SVM)
Algarni, et al. [21]	2022	Bi-LSTM	DEAP	96.87%, 99.45%, 99.68% (**D**; Arousal, Valence, liking)	+11.42%, +13.80%, +11.69 (**D**; Arousal, Valence, liking; vs. LSTM-RNN)
Shen et al. [78]	2023	CLISA	THU-EP, SEED	subject-independent: 2-categories: 71.9 ± 8.8% (**T**) 9-categories: 45.7 ± 11.8% (**T**) 86.4± 6.4% (**S**)	subject-independent: 2-categories: +6.7% (**T**; vs. DE + MLP) 9-categories: +10.4% (**T**; vs. DE + MLP) +6.5% (**S**; vs. DE + MLP)
Ding, et al. [36]	2023	TSception	DEAP, MAHNOB-HCI, DEAP	61.57% ± 11.04%, 59.14% ± 7.60% (**D**; Arousal, Valence) 60.61% ± 14.88%, 61.27% ± 10.05% (**MA**; Arousal, Valence) 63.75%, 62.27% (**D**; Arousal, Valence)	+1.20%, +3.95% (**D**; Arousal, Valence; vs. SVM) +2.36%, +2.83% (**MA**; Arousal, Valence; vs. SVM) +1.75%, +4.67% (**D**; Arousal, Valence; vs. SVM)
Jang, et al. [47]	2023	GNN	DEAP, DREAMER	subject-dependent: 73.5% ± 8.07% (**D**) 55.5% ± 7.59% (**DR**)	subject-dependent: +69.95% (**D**; vs. SVM) +36.20% (**DR**; vs. SVM)
Liu, et al. [53]	2024	MAS-DGAT-Net	SEED, SEED-IV, DEAP	subject-dependent: 99.86% ± 1.12% (**S**) 99.53% ± 1.63% (**S4**) 95.21% ± 4.52%, 94.69% ± 4.11% (**D**; Arousal, Valence) subject-independent: 99.86% ± 1.12% (**S**) 99.53% ± 1.63% (**S4**) 62.86% ± 5.34%, 64.76% ± 5.02% (**D**; Arousal, Valence)	subject-dependent: +15.87% (**S**; vs. SVM) +42.92% (**S4**; vs. SVM) +39.41%, +43.09% (**D**; Arousal, Valence; vs. SVM) subject-independent: +19.91% (**S**; vs. DGCNN) +46.71% (**S4**; vs. DGCNN) +9.18%, +13.44% (**D**; Arousal, Valence; vs. DGCNN)
Chen, et al. [54]	2024	GDDN	SEED, SEED-IV, MPED	subject-independent: 92.54% ± 3.56% (**S**) 75.65% ± 5.47% (**S4**) 29.44% ± 3.80% (**M**) cross-dataset: 63.7% (**S→S4**), 59.15% (**S4→S**), 42.36% (**S→M**), 65.51% (**M→S**)	subject-independent: +35.81% (**S**; vs. SVM) +36.26% (**S4**; vs. SVM) +9.78% (**M**; vs. SVM) cross-dataset: +13.10% (**S→S4**; vs. IAG), +7.17% (**S4→S**; vs. IAG), +8.94% (**S→M**; vs. SVM), +32.98% (**M→S**; vs. SVM)
Tang, et al. [64]	2024	MD^2^GRL	SEED, DEAP	subject-dependent: 99.23% ± 1.74% (**S**) 95.77% ± 2.35%, 96.51% ± 2.89% (**D**; Arousal, Valence) subject-independent: 96.23% ± 2.57% (**S**) 92.04% ± 3.96%, 92.58% ± 3.80% (**D**; Arousal, Valence)	subject-dependent: +8.83% (**S**; vs. DGCNN) +9.56%, +9.58% (**D**; Arousal, Valence; vs. DGCNN) subject-independent: +11.78% (**S**; vs. DGCNN) +31.48%, +32.94% (**D**; Arousal, Valence; vs. DGCNN)
Zhang, et al. [66]	2025	CI-Graph	SEED, SEED-IV, DEAP	subject-independent: 88.96% ± 4.68% (**S**) 76.23% ± 8.15% (**S4**) 65.84% ± 10.36%, 62.93% ± 6.95% (**D**; Arousal, Valence)	subject-independent: +4.16% (**S**; vs. DGCNN) +5.98% (**S4**; vs. DGCNN) +1.13%, +1.28% (**D**; Arousal, Valence; vs. DGCNN)
Shen et al. [79]	2025	DAEST	FACED, SEED, SEED-IV	subject-independent: 2 categories: 81.7 ± 4.3% (**F**) 9 categories: 67.9 ± 7.3% (**F**) 88.1 ± 3.6% (**S**) 73.6 ± 12.7% (**S4**)	subject-independent: 2 categories: +12.9% (**F**; vs. CLISA) 9 categories: +25.5% (**F**; vs. CLISA) +2.5% (**S**; vs. CLISA) +8.9% (**S4**; vs. CLISA)

* Dataset abbreviation (bold in the table): S: SEED; S4: SEED-IV; M: MPED; MA: MAHNOB-HCI; D: DEAP; DR: DREAMER; F: FACED; T: THU-EP.

**Table 2:** Sample use of minimal C-I qualitative assessment heuristic.

Method (Year)	Commonality Gain	Individuality Preserved	Explicit C–I Trade-Off
Multi-Column CNN (2019) [24]	Δ	√	× (Not applied)
IAG (2020) [59]	√√	√√	Δ (implicit balance)
MSDA-SFE (2023) [74]	√√	Δ	√ (domain-invariance & domain-specific feature learning module)
CLISA (2023) [78]	√√	Δ	√ (Contrastive alignment)
CI-Graph (2025) [66]	√√	√√	√√ (jointly optimize both commonality & individuality branches)

Symbols: √√/√ = strong/present, Δ = weak, × = absent.

## Data Availability

No new data were created or analyzed in this study.
